# Surface-Based Body Shape Index and Its Relationship with All-Cause Mortality

**DOI:** 10.1371/journal.pone.0144639

**Published:** 2015-12-28

**Authors:** Syed Ashiqur Rahman, Donald Adjeroh

**Affiliations:** Lane Department of Computer Science and Electrical Engineering, West Virginia University, Morgantown, WV 26506, United States of America; Peking Union Medical College, CHINA

## Abstract

**Background:**

Obesity is a global public health challenge. In the US, for instance, obesity prevalence remains high at more than one-third of the adult population, while over two-thirds are obese or overweight. Obesity is associated with various health problems, such as diabetes, cardiovascular diseases (CVDs), depression, some forms of cancer, sleep apnea, osteoarthritis, among others. The body mass index (BMI) is one of the best known measures of obesity. The BMI, however, has serious limitations, for instance, its inability to capture the distribution of lean mass and adipose tissue, which is a better predictor of diabetes and CVDs, and its curved (“U-shaped”) relationship with mortality hazard. Other anthropometric measures and their relation to obesity have been studied, each with its advantages and limitations. In this work, we introduce a new anthropometric measure (called Surface-based Body Shape Index, SBSI) that accounts for both body shape and body size, and evaluate its performance as a predictor of all-cause mortality.

**Methods and Findings:**

We analyzed data on 11,808 subjects (ages 18–85), from the National Health and Human Nutrition Examination Survey (NHANES) 1999–2004, with 8-year mortality follow up. Based on the analysis, we introduce a new body shape index constructed from four important anthropometric determinants of body shape and body size: body surface area (BSA), vertical trunk circumference (VTC), height (H) and waist circumference (WC). The surface-based body shape index (SBSI) is defined as follows:

$$SBSI=\frac{\left(H^{7/4}\right)\left(WC^{5/6}\right)}{BSA\;VTC}$$ (1)

SBSI has negative correlation with BMI and weight respectively, no correlation with WC, and shows a generally linear relationship with age. Results on mortality hazard prediction using both the Cox proportionality model, and Kaplan-Meier curves each show that SBSI outperforms currently popular body shape indices (e.g., BMI, WC, waist-to-height ratio (WHtR), waist-to-hip ratio (WHR), A Body Shape Index (ABSI)) in predicting all-cause mortality.

**Conclusions:**

We combine measures of both body shape and body size to construct a novel anthropometric measure, the surface-based body shape index (SBSI). SBSI is generally linear with age, and increases with increasing mortality, when compared with other popular anthropometric indices of body shape.

## Introduction

Obesity, with its dual complications of diabetes mellitus and cardiovascular disease (CVD), has emerged as a major public health challenge [1–3]. Obesity is identified by the World Health Organization (WHO) as a global epidemic [4]. In the US, obesity prevalence remains high at 35.7% of the adult population [5], while 68% are classified as obese or overweight [1], with the highest rates being found among the populations that are poor, have lower education, and are minority groups [1]. The picture for childhood and adolescent obesity is no better, with 16.9% obesity prevalence, and 31.8% classified as obese or overweight [5], and thus at the risk of developing insulin resistance, dyslipidemia, or hypertension at an early age [6]. This trend is mirrored by the high incidence of diabetes, which has shown a similarly high prevalence rates [7]. The problem of obesity is attributed to the issue of imbalance between energy intake and energy expenditure in the body [8]. The problem is directly connected to the quantity of adipose depots (body fat). Adiposity is associated with increased risk of many chronic diseases in the general population [9–13]. Obesity is known to be associated with diabetes, and various forms of cardiovascular disease (CVD). Other associated complications include depression, mobility issues, some forms of cancer [14], sleep apnea [15], osteoarthritis, among others (see [8] for a review).

Many different anthropometric measures have been used to assess adiposity. The body mass index (BMI) is one of the best known indices of relative adiposity or excess body weight in the association of body composition with mortality. Individuals are often grouped into BMI categories [4, 16] (underweight (*BMI* < 18.5), normal weight (18.5 ≤ *BMI* < 25), overweight (25 ≤ *BMI* < 30), obese I (30 ≤ *BMI* < 35), obese II (35 ≤ *BMI* < 40), and obese III (*BMI* ≥ 40)). Risk of CVD and diabetes tends to increase with increasing BMI. The association of BMI with mortality in the general population is usually found to exhibit a U-shaped [17, 18] or J-shaped [19, 20] curve. Using BMI-defined categories Flegal et al [18] showed, that obese and underweight individuals had a higher death rate, while normal weight and overweight individuals had a similar relative mortality risk. Some have hypothesized that the non-linear relationship observed between BMI and mortality may be a consequence of BMI being a composite of both fat and fat-free mass [21, 22], not simply a surrogate for overall adiposity. These observations point to the core limitation of BMI as a measure of adiposity. Several studies have shown that adjustment for waist circumference, a surrogate for abdominal adiposity [23–25], eliminates or attenuates BMI’s nonlinear relationship with mortality [26, 27]. The ABSI (A Body Shape Index, defined as *ABSI* = *WC*/(*BMI* ^2/3^ * *H* ^1/2^) which places more emphasis on waist circumference was proposed by Krakauer and Krakauer [28] as an alternative to the BMI, resulting in a better prediction of mortality hazard. Various other one dimensional (1D) anthropometric measures (e.g., waist circumference (WC), hip circumference (HC), skin folds (SFs)), and their relation to obesity have also been studied. Example, the waist-to-hip ratio (WHR) is a better indicator of ischemic heart disease mortality [29], while the waist-to-height ratio (WHtR) provides a better predictor for death, heart attack and stroke [30]. Ohrvall et al [31] and Pouliot et al [32], showed sagittal abdominal diameter to be a better measure of the accumulation of visceral adipose tissue and cardiovascular risk. Beyond 1D measures, there are also studies linking obesity-related diseases with 2D measures (e.g., body surface area (BSA) [33]), and 3D measures [34–36], (e.g., body volume index (BVI) [22, 37]).

Results in [28] showed that ABSI produced better results than both BMI and WC in terms of all-cause-mortality hazard prediction. More recent studies, however, show that ABSI does not perform better than WC for diabetes mellitus (DM) prediction [38]. He et al [38], showed that for the Chinese population, the three measures WC, BMI, and ABSI showed similar predictive abilities. Zhang et al [39], showed ABSI to be a weak predictor for the risk of cardiovascular diseases (CVD), or the problem of Metabolic Syndrome (MetS). Clearly, no single measure can capture all aspects of the general problem of obesity and its related diseases. In this work, we first introduce a new anthropometric measure (called Surface-based Body Shape Index, SBSI) that accounts for both body shape and body size. Then, we evaluate the proposed measure as a predictor of all-cause-mortality, and compare its performance with other popular body shape indices, namely BMI, WC, and ABSI.

## Materials and Methods

### Datasets

We used mortality data combined with anthropometric data from the National Health and Human Nutrition Examination Surveys (NHANES) 1999–2004 [40–42]. NHANES employs a complex cluster design to sample members of the civilian USA population who are not institutionalized. NHANES uses stratified multistage probability to sample the data. Mortality information from public-use mortality files is linked to the National Death Index (NDI). Since not all the data contained mortality information we excluded those individuals that do not have data on mortality. Ethnicity included white, black, Mexican and others. Anthropometric measurements included BMI, height, weight, and waist circumference. We used the NHANES mobile examination center sample. The mobile examination center used trained examiners who used standardized protocols to measure the anthropometric parameters. Mortality data based on NDI were available in 2006. After refining, we obtained 11,808 individuals with 701 deaths during the 2–8 years of follow-up (1999–2006).

We also used data from the Civilian American and European Surface Anthropometry Resource (CAESAR) [43]. CAESAR project was a survey of the civilian populations from four countries namely the United States of America (USA), Canada, the Netherlands, and Italy. The survey was carried out by the U.S. Air Force, resulting in complete 3-D models of each civilian subject. The 3-D surface anthropometry was performed using three scanned poses using the cyberware 3D whole-body scanner [44]. The CAESAR dataset also includes manual hand measurements of the various anthropometric attributes, recorded as 1D information. For our purpose, we used the 1D datasets from the CEASAR survey, which contains 2400 US and Canadian civilians, ages 18–65 (http://store.sae.org/caesar/). We selected 45 key human body measurements, as reported by Adjeroh et al [45] and Cao et al [46]. From our analysis, the key measurements shared by both datasets tend to have similar general statistics. For example, the mean and standard deviation were observed as follows: height (NHANES 167.7 ± 10.1; CAESAR 170.46 ± 10.2), waist circumference (NHANES 92.2 ± 13.2; CAESAR 84.8 ± 14.4), weight (NHANES 74 ± 15.8; CAESAR 77 ± 19.8), BMI (NHANES 26.2 ± 4.7; CAESAR 26.3 ± 5.7). Apart from the prediction of VTC for NHANES samples based on learned parameters from CAESAR, all other analyses are based on the NHANES dataset.

### Study Variables

In the CAESAR study [43], data collection was a three-step process: in-processing/demographics; traditional hand measurements with tape and calipers; and 3D whole-body scanning stations. All measurements were taken with participants wearing light clothes and without shoes. For height, subject stands fully erect with weight distributed equally on both feet, with both arms hanging freely downwards. Thigh circumference was measured on a seated subject. Triceps skinfold is the thickness of the skinfold overlaying the triceps muscle. This was measured on the back of the upper arm, between the tip of the shoulder and the elbow while the subject’s arm is bent 90°. VTC was measured using a tape from the shoulder, through the crotch, and back to the shoulder while the subject stands fully erect with the weight distributed equally on both feet and the arms hanging freely downwards. Waist circumference is the maximum circumference of the waist that can be measured using a tape measure, which starts at the top of subject’s hip bone, then all the way around level with his/her belly button. Height, circumferences, and length measurements were made to the nearest 0.1 cm, while weight was measured to the nearest kilogram.

In the NHANES study [40–42], anthropometric measurements were taken by trained personnel. Height was obtained using a digital meter. Subjects wore a light examination gown before measuring their weight on a digital scale. Waist circumference was measured just above the uppermost lateral border of the ilium. A key component of the proposed SBSI is the vertical trunk circumference (VTC). Given that NHANES does not contain information on the VTC for its subjects, we first learned the regression parameters for predicting the VTC using the CAESAR dataset. Then, we applied the learned parameters on the samples from NHANES to predict their VTC. Since the two datasets have similar overall statistics, we can rely on the results of the prediction for subjects in NHANES. Based on the measures, we computed the BMI using the standard formula: *BMI* = *W*/*H* ^2^ (unit *kg*/*m* ^2^), where W is weight (kg), and H is height (m). The body surface area (BSA) is assessed following Shuter and Aslani [47]: *BSA* = 0.00949 × *W* ^0.441^ × *H* ^0.655^. BMI obesity categories were computed following WHO definitions [56, 62]: underweight (*BMI* < 18.5), normal (18.5 ≤ *BMI* < 25), overweight (25 ≤ *BMI* < 30), obese I (30 ≤ *BMI* < 35), obese II (35 ≤ *BMI* < 40), and obese III (*BMI* ≥ 40).

### Surface-based Body Shape Index (SBSI)

The BMI provides a simple coarse measure of the body shape. Two people in the same BMI category could have very different body shapes, and different body sizes. The distribution of body weight, rather than the absolute weight, is a key factor in predicting health risk. A person with much of the body weight around the midsection is at a much greater risk of disease and early mortality, when compared with another person that has weight better distributed peripherally (especially in lower body) [48]. This observation relates to the so-called ‘apple-shaped vs. pear-shaped’ phenomena, whereby the waist-to-hip ratio (WHR) is used to determine whether a person is apple-shaped (*WHR* < 0.8 for women, *WHR* < 0.9 for men), or pear-shaped (*WHR* ≥ 0.8 for women, *WHR* ≥ 0.9 for men). See [48]. The waist circumference (WC) is often combined with the BMI for an improved assessment of body shape [28]. Other studies used waist-to-height ratio (WHtR) as a shape index [49]. In addition to body shape, the body size is also another important factor. While indices such as BMI, ABSI, waist-to-height ratio (WHtR) measure body shape, others such as BSA, WC, H, and VTC provide some indication of body size. The body surface area (BSA) provides a measure of the body size, while the VTC measures both the body size, and body shape. In this work, we consider both body shape and body size simultaneously, and thus combine the BSA and VTC with height and WC to develop a new surface-based body shape index.

**Fig 1:**
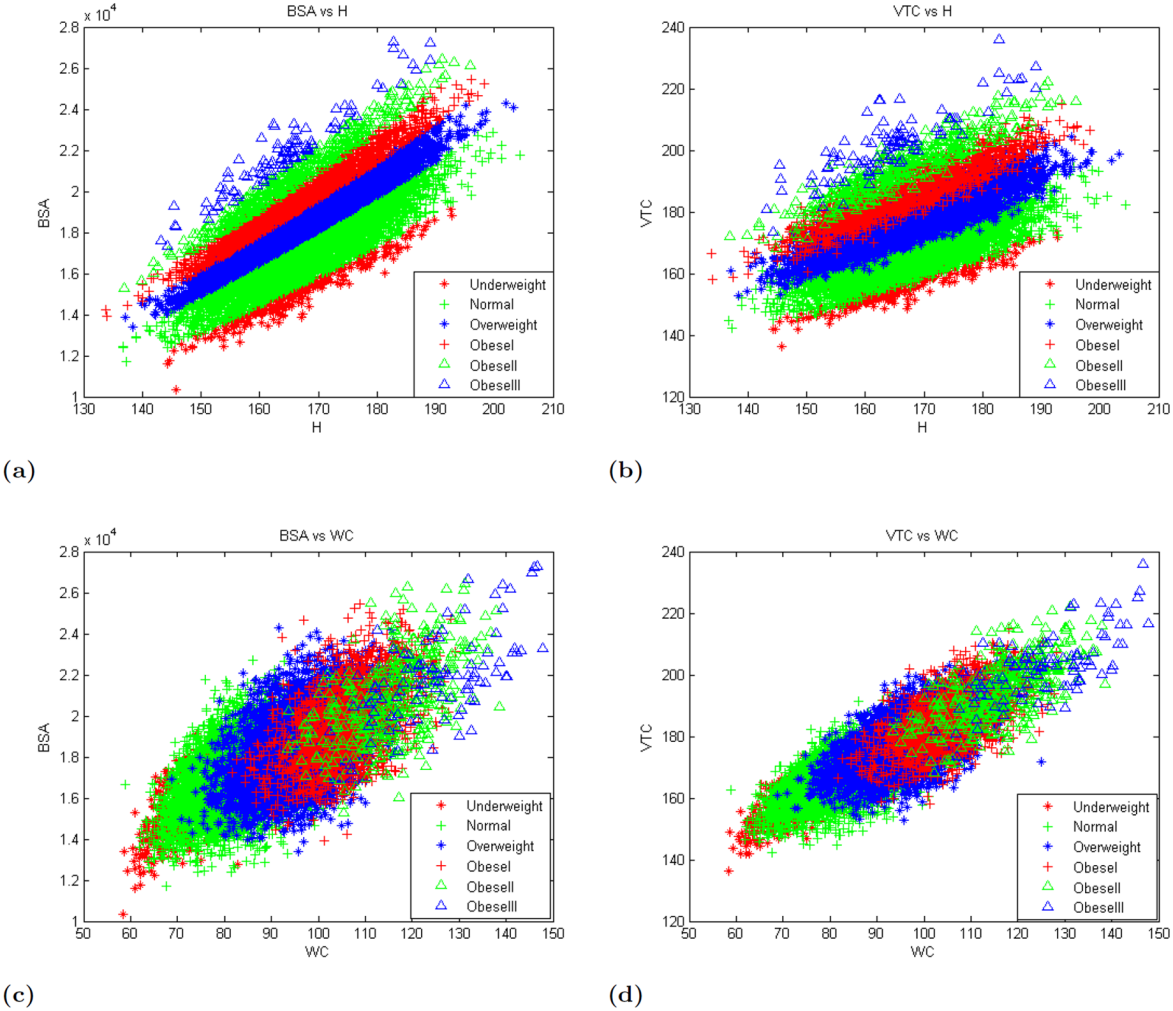
Relationship between BSA, VTC, height and WC for given BMI categories. The BSA and height, and VTC and height can predict the BMI categories (a, b). BSA and WC (and VTC and WC) show a non-linear relationship for a given BMI category (c, d).

To investigate the significance of the BSA and VTC, we analyzed their relationship with height and waist circumference, for a given BMI category, using the NHANES dataset. The results are shown in Fig 1. It can be observed that, at a given height, obese individuals tend to have higher BSA, while those that are underweight tend to have a lower BSA. At a given height, the BSA tends to increase steadily with BMI (Fig 1a). VTC and height show a similar behavior at given BMI categories (Fig 1b). The relationship between BSA and WC (or VTC and WC) is not as clear. Unlike the clear linear association between BSA (or VTC) and height, for a given BMI category, BSA (VTC) has a non-linear relationship with WC, for a given BMI. Yet, the different BMI categories are evident from the graphs (Fig 1c and 1d). The underweight group clustered in mainly the bottom left quadrant, while the obese III category clustered around the top right quadrant.

### SBSI Construction

Different formulae have been proposed for estimating the body surface area (BSA), based mainly on the weight and height. In a survey of different BSA predictors [50], the Shuter and Aslani method [47] was shown to provide an overall best performance. Thus, in this work, we adopt this method to predict BSA as follows:

$$BSA=0.00949\times W^{0.441}\times H^{0.655},$$ (2)

where W = weight in kilograms, and H = height in meters. For VTC, we first identified common anthropometric measurements between the CAESAR and NHANES datasets. Then we performed simple linear regression using the samples in CAESAR and subsequently applied regression learning to predict the VTC for the samples in the NHANES database. The VTC (in cm) is predicted using the formula:

$$\begin{array}{cc} VTC= & (61.2\pm 2.81)+AC(0.315\pm 0.03)+H(0.409\pm 0.01)+SS(0.237\pm 0.13)-\\ & TC(0.089\pm 0.02)-TS(0.12\pm 0.13)-UAL(0.453\pm 0.05)+WC(0.137\pm 0.01)+\\ & W(0.37\pm 0.02), \end{array}$$ (3)

where AC = arm circumference (cm), H = height (cm), SS = subscapular skin fold (cm), TC = thigh circumference (cm), TS = triceps skinfold (cm), UAL = upper arm length (cm), WC = waist circumference (cm), W = weight (kg). This prediction resulted in error measure (*R* ^2^ = 0.9217, p-value (P) = 2.2 × 10^−16^) using the CAESAR dataset.

We then combine the BSA and VTC with the height and waist circumference by using linear regression on log(*BSA* × *VTC*) as a function of log(*H*) and log(*WC*) for the entire database:

log(BSA×VTC)=(0.922±0.02)+(1.766±0.009)log(H)+(0.838±0.003)log(WC) (4)

The error measure for this prediction was (*R* ^2^ = 0.9, *P* = 2.2 × 10^−16^). From the above, we can infer the relationship:

$$\left(BSA\times VTC\right)\propto \left(H^{7/4}\right)\left(WC^{5/6}\right)$$ (5)

Taking ratios of the two sides, we define the Surface-based Body Shape Index (SBSI) as follows:

$$SBSI≜\frac{(H^{7/4)}\left(WC^{5/6}\right)}{BSA\;VTC}$$ (6)

### Statistical Analysis

We analyzed the data separately for male and female subjects, and for their combination. Table 1 shows the characteristics of the study participants for NHANES dataset. The corresponding data for the CAESAR dataset is provided as Supplementary Material in S1 Table. The sample mean for SBSI (using NHANES) is 0.10718±0.00627, with a minimum of 0.08218, and a maximum of 0.14228. For CAESAR dataset, we observed mean SBSI of 0.10644±0.006092, a minimum of 0.07437, and a maximum of 0.1386.

**Table 1:** Key anthropometric attributes for study participants.

	**All (N = 11808)**		**Female (N = 5840)**		**Male (N = 5968)**	
	Average	SD	Average	SD	Average	SD
Age(years)	45.692	20.726	45.418	20.920	45.959	20.532
Weight(W)(kg)	74.015	15.801	67.905	14.065	79.994	15.105
Height(H)(cm)	167.726	10.102	160.936	7.136	174.371	7.925
BMI(*kg*/*m* ^2^)	26.227	4.696	26.201	5.065	26.253	4.306
Leg Length(cm)	40.561	3.790	38.638	3.217	42.443	3.337
Arm Length(cm)	37.088	2.731	35.489	2.137	38.654	2.309
Arm Circumference(cm)	31.262	4.072	30.084	4.102	32.415	3.694
Waist Circumference(cm)	92.190	13.229	89.573	13.035	94.752	12.914
Thigh Circumference(cm)	51.362	5.984	50.921	6.346	51.793	5.573
Triceps Skinfold(cm)	17.675	8.066	22.387	7.235	13.064	5.875
Subscapular skinfold(cm)	19.072	7.566	20.049	7.848	18.116	7.152
Vertical Trunk Circumference(VTC)(cm)	174.924	11.908	170.349	10.845	179.402	11.177
A Body Shape Index(ABSI)(*m* ^11/6^ *kg* ^−2/3^)	0.081	0.005	0.080	0.006	0.081	0.005
Body Surface Area(BSA)(*cm* ^2^)	18074	2168	16927	1759	19196	1930
WHtR	0.551	0.080	0.557	0.084	0.544	0.075
Surface Based Body Shape(SBSI)	0.107	0.006	0.107	0.007	0.108	0.006

(using the NHANES dataset.)

Table 2 shows the correlation between the SBSI and other anthropometric body indices. The table shows the correlation using direct measurements for both Pearson’s *ρ* (upper half), and Kendall’s *τ* (lower half). For a given measurement value *x*, its *z*-score is computed as *z*(*x*) = (*x* − *μ*)/*σ*, where *μ* and *σ* are the mean and standard deviation for the measurement. SBSI has high correlation with ABSI, low correlation with WC and height, and negative correlation with BMI and weight. The reason $ABSI=\frac{WC}{\left(BMI^{2/3}\times H^{1/2}\right)}$ has a high correlation with SBSI might be because of the fact that both ABSI and SBSI use WC, and H in similar roles in their respective formulae.

**Table 2:** Correlation coefficient between anthropometric measures.

	H	W	BMI	WC	VTC	BSA	ABSI	SBSI
H	1	0.548	-0.023	0.194	0.568	0.761	-0.007	-0.025
W	0.385	1	0.817	0.826	0.975	0.958	0.079	−0.406
BMI	-0.005	0.612	1	0.855	0.778	0.626	0.091	-0.475
WC	0.129	0.620	0.668	1	0.838	0.708	0.542	-0.023
VTC	0.390	0.864	0.576	0.634	1	0.948	0.163	-0.357
BSA	0.561	0.824	0.435	0.499	0.791	1	0.062	-0.323
ABSI	0.006	0.072	0.093	0.391	0.125	0.055	1	0.801
SBSI	-0.011	-0.251	-0.293	0.003	-0.218	-0.197	0.583	1

Pearson’s *ρ* (upper half), and Kendall’s *τ* (lower half).

We used Cox proportionality mortality hazard modeling [51, 52] to quantify the association of the proposed SBSI and other anthropometric measures (ABSI, BMI, and WC) with all-cause mortality. Under the Cox model, the relationship between hazard and the covariates is described by considering the logarithm of the hazard as a linear function of the variables. Following the Poisson model, this can be expressed by using exponentiation on the covariate terms [52]:

$$h\left(t,x\right)=\exp\left(\beta_{0}+\beta_{1}x\right)=h_{0}\exp\left(\beta_{1}x\right)>0$$ (7)

where, *h*_0_ is the baseline hazard, *β*_0_ and *β*_1_ are coefficients influencing the covariates *x*. This is often generalized as follows:

$$h\left(t,x\right)=h_{0}\left(t,\alpha \right)\exp\left(\beta^{T},x\right)$$ (8)

where *α* are the parameters influencing the baseline hazard. In our approach we modeled the log death rate as a nonparametric function of time (months of follow-up from the interview) and coefficients are fitted which multiply the value of the predictor variables. Although predictors can be entered as either continuous or discrete, we used predictor’s *z*-score as continuous variables for generalization. Previous studies suggest that using *z*-score in the hazard model produce better results [28]. We calculated mortality risk associated with each anthropometric measurement separately for male and female subjects, and later for all subjects in the dataset. Then we divided the dataset using BMI categories to test the range of applicability of our proposed SBSI and also how it compares with other existing body shape indices. We used the *R* ^2^ statistic to measure how successful the model is in explaining the variation of the data.

To further study the predictive capabilities of SBSI and to compare with other body shape indices, we constructed and analyzed the Kaplan-Meier (KM) curves [53] using each measure. The Kaplan-Meier estimate of the survival function is a non-parametric method of estimating survival from data. It is very popular because it makes only very weak assumptions about the data. In medical research, it is used to measure the fraction of patients surviving for a certain amount of time after treatment. Let *S*(*t*) be the probability that a member from a given population will have a lifetime exceeding t. For a sample of size *N* from this population, let the observed times until death of the *N* sample members be *t*_1_ ≤ *t*_2_ ≤ *t*_3_ ≤ … ≤ *t*_*N*_. Corresponding to each *t*_*i*_ is *n*_*i*_, the number “at risk” just prior to time *t*_*i*_, and *d*_*i*_, the number of deaths at time *t*_*i*_. The Kaplan–Meier estimator is the nonparametric maximum likelihood estimate of $\hat{S}\left(t\right)$, where $\hat{S}\left(t\right)$ is a product of the form

$$\hat{S}\left(t\right)=\prod_{t_{i}\leq t}\frac{n_{i}-d_{i}}{n_{i}},$$ (9)

We performed analysis using KM survival curve estimates for all the data, and separately for all female, and all male. Then we did more rigorous study based on BMI categories. We used the log-rank test to compare the survival distributions obtained using different shape indices. The log-rank test tries to distinguish between Kaplan-Meier curves to see if they are statistically equivalent. The output of the test is a *χ* ^2^-distance, and the P-value associated with the distance. Higher *χ* ^2^-distances and low P-values indicate a better separation between the curves, and hence a better performance in mortality modeling. All statistical analyses were performed using the R Language (ver. 3.0.3, The R Foundation for Statistical Computing, Vienna, Austria). We considered *P* ≤ 0.05 to be statistically significant.

## Results

### Higher SBSI with Increasing Age

**Fig 2:**
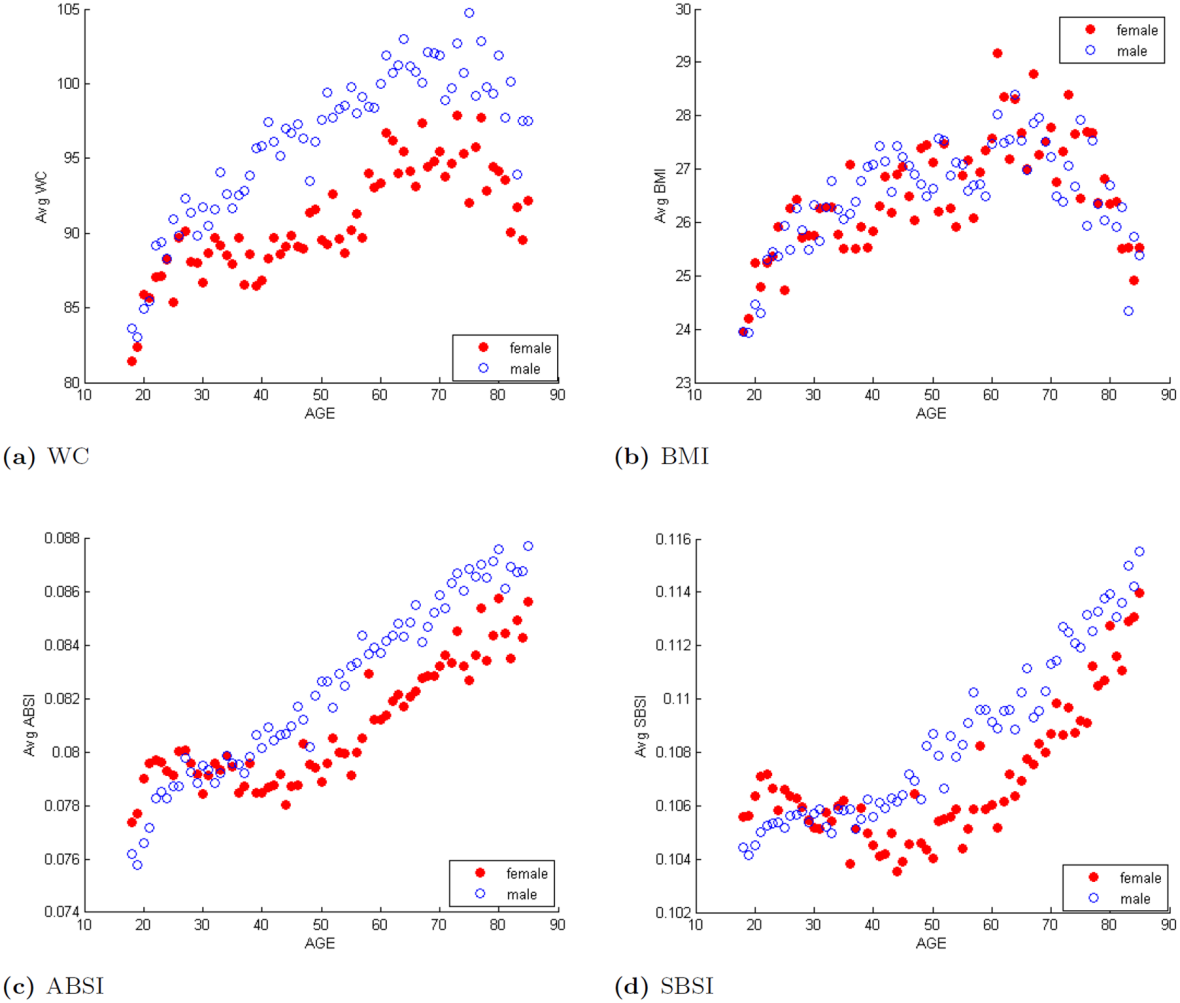
Variation of different body shape indices with age (in years).

The SBSI increases generally with increasing age. Fig 2 shows how the SBSI and three other anthropometric measures vary with age for both male and female. Subjects in our NHANES dataset had age ranges between 18–85 years. Mean SBSI increases consistently (generally linearly) for male subjects. However, for female subjects, the mean goes up from about ages 18 to 24 then goes down till about age 30; between ages 30 and 40, the mean SBSI did not have a definite pattern for females, going up and then down. After this point, the SBSI generally increased with age. For WC, ABSI and SBSI, the mean values for males were clearly separated from those for females, with the value for males being generally greater. For the BMI, this distinction was not as clear. Unlike ABSI and SBSI that had a generally linear relationship with age, the BMI and WC had an inverted U-like shape, with the turning points around age 75 for WC, and age 70 for BMI.

### Higher Mortality Hazard for Increasing SBSI

**Fig 3:**
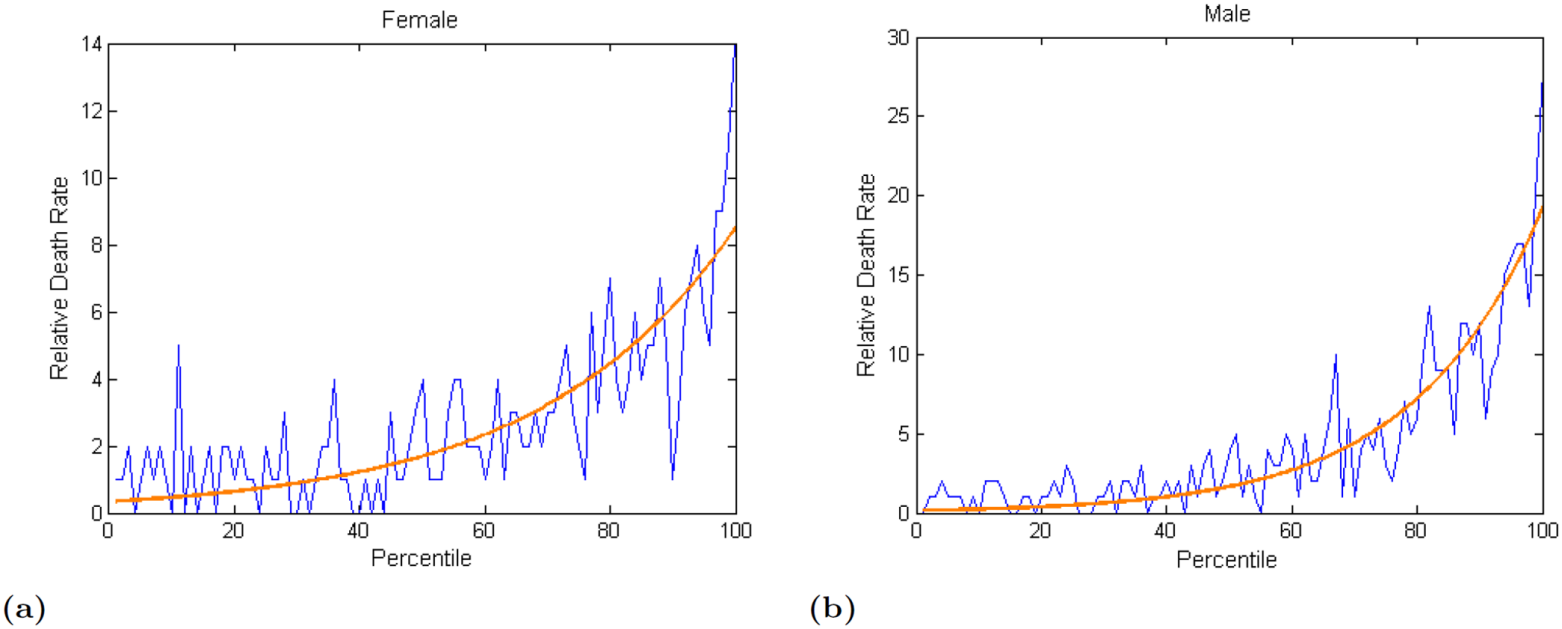
Variation of relative death rate with increasing values of SBSI z-score. (a) Female; (b) Male.

Fig 3 shows the variation of the relative death rate with SBSI using their *z*-scores. The figure shows that the relative death rate increases almost exponentially with increasing values of SBSI. The variability in the mortality hazard prediction also seems to increase with increasing SBSI values. The results in this figure are consistent with known results that relative death rate is generally higher for male than female subjects. In Fig 3(a) the relative death rate for female was almost similar until about the 50th percentile (average 1.3) then it went up (from 4 to 14). For male (Fig 3b) average death rate was 1.02 until about the 35th percentile, after that it grew exponentially (from 3 to 28).

### Improved Modeling for All-Cause Mortality using SBSI

The proposed surface-based body shape index shows substantial improvements in mortality modeling, when compared with popular body shape indices. Table 3 shows the summary performance in mortality hazard modeling for SBSI, ABSI, BMI, and WC. The hazard ratio (HR) for SBSI was 2.287 for all, 2.019 for female and 2.456 for male. For all measures, the results are based on using their *z*-scores as a continuous variable, rather than the original value.

**Table 3:** Summary of mortality hazard ratio for four key anthropometric measures.

	*ALL*	*Female*	*Male*
*BMI*	0.915 (0.849 – 0.987)	1.019 (0.9148 – 1.136)	0.823 (0.738 – 0.9172)
*WC*	1.327 (1.237 – 1.425)	1.338 (1.195 – 1.498)	1.254 (1.14 – 1.378)
*ABSI*	2.328 (2.173 – 2.495)	1.999 (1.8 – 2.22)	2.682 (2.439 – 2.951)
*SBSI*	2.287 (2.142 – 2.443)	2.019 (1.809 – 2.253)	2.456 (2.269 – 2.658)

Results are reported as HR (95% CI).

**Fig 4:**
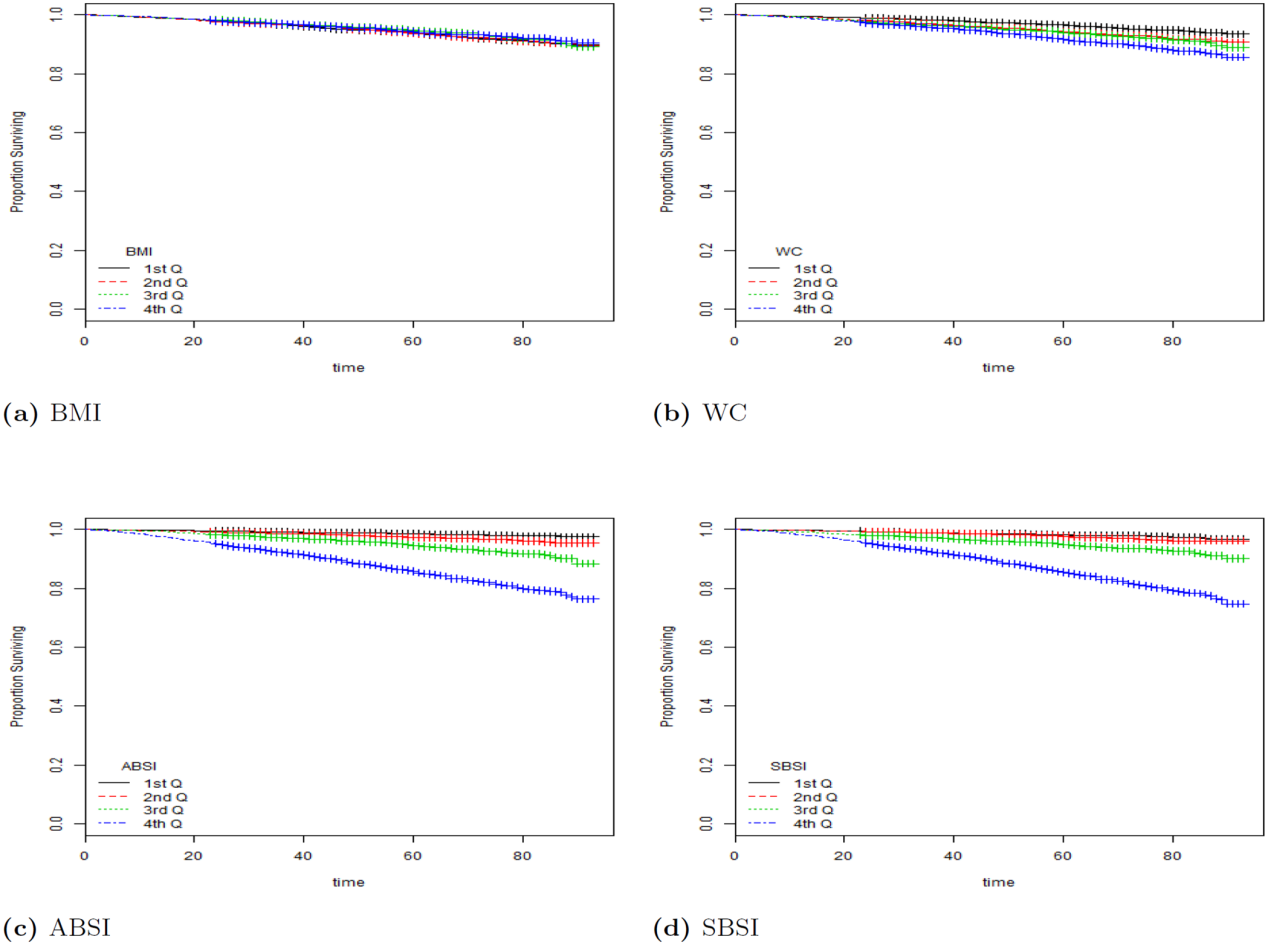
The Kaplan Meier curves for four body shape indices using all subjects. The SBSI shows a better prediction performance than other body shape measures (with more separation between the curves, and less crossovers). 1st Q, 2nd Q, etc. denote respectively 1st quartile, 2nd quartile, etc.

Table 4 shows the corresponding results in terms of the *χ* ^2^-distance when using the logrank test to analyze the KM survival curves for each body shape index. The *χ* ^2^-distance for SBSI was 570 for all, 147.68 for female and 434.372 for male. Here the analyses was done on the quartiles labeled as 1st Q, 2nd Q, etc. in Fig 4. From the table, SBSI performs significantly better than waist circumference and ABSI. Clearly, the BMI was unable to show a distinction in the survival rates for the quartiles, given its non-linear relationship with mortality-hazard.

**Table 4:** Summary of *χ* ^2^-distances for KM survival curves for four key anthropometric measures.

	*ALL*	*Female*	*Male*
*BMI*	2.20 (0.531)	1.019 1.345 (0.718)	6.064 (0.108))
*WC*	59.464(7.6510^−13^)	22.506(5.1210^−5^)	25.961(9.7210^−6^)
*ABSI*	551.126 (0)	141.697 (0)	415.643 (0)
*SBSI*	570.044 (0)	147.688 (0)	434.372 (0)

Results are reported as *χ* ^2^-distance (P-value).

**Fig 5:**
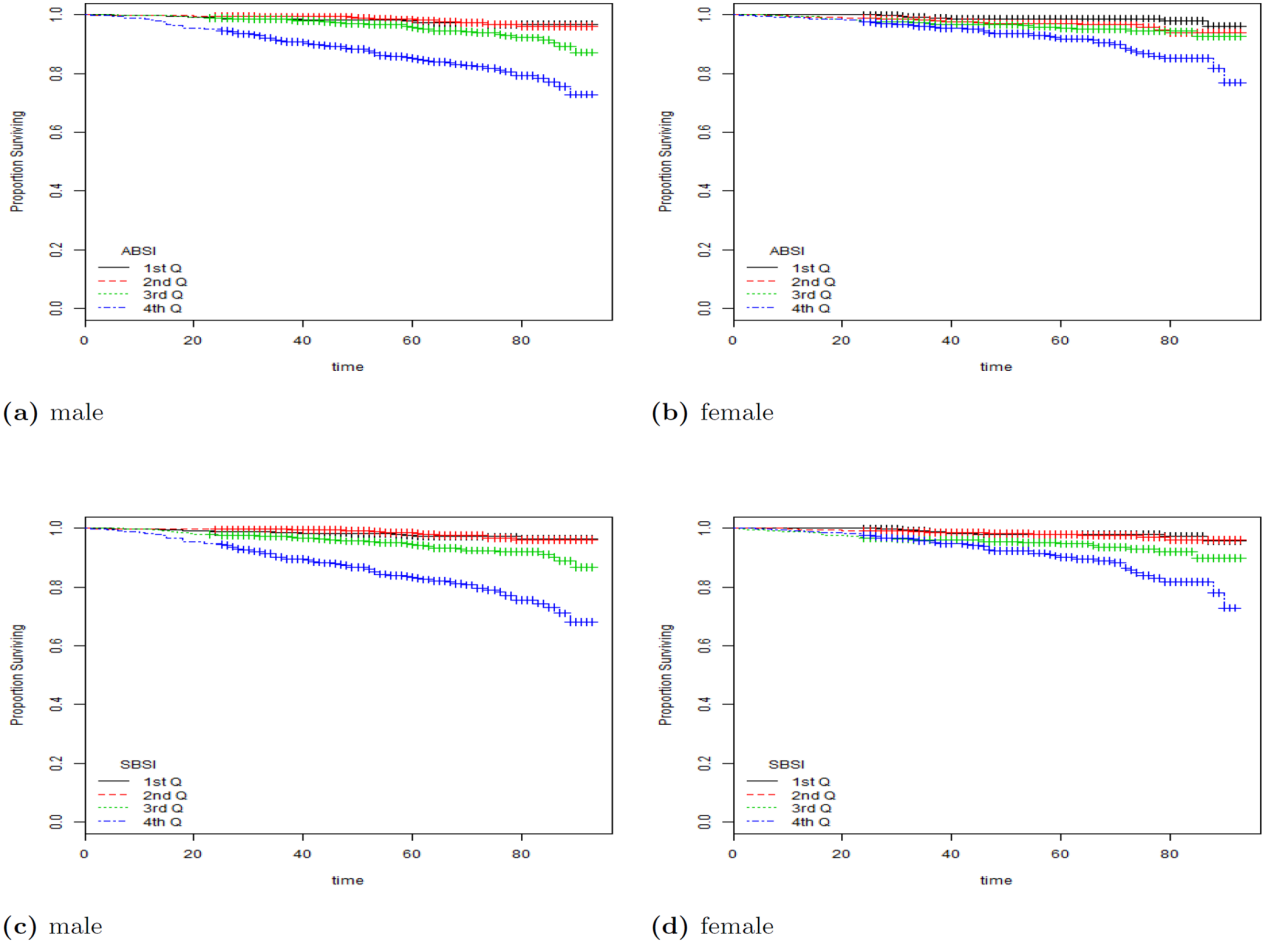
The KM curves using ABSI and SBSI on subjects in the BMI category overweight. a: ABSI (male); b: ABSI (female); c: SBSI (male); d: SBSI (female). As expected, both measures indicate that female subjects have better survival rates when compared with male subjects. SBSI shows an overall better prediction performance than ABSI. 1st Q, 2nd Q, etc. denote respectively 1st quartile, 2nd quartile, etc.

**Fig 6:**
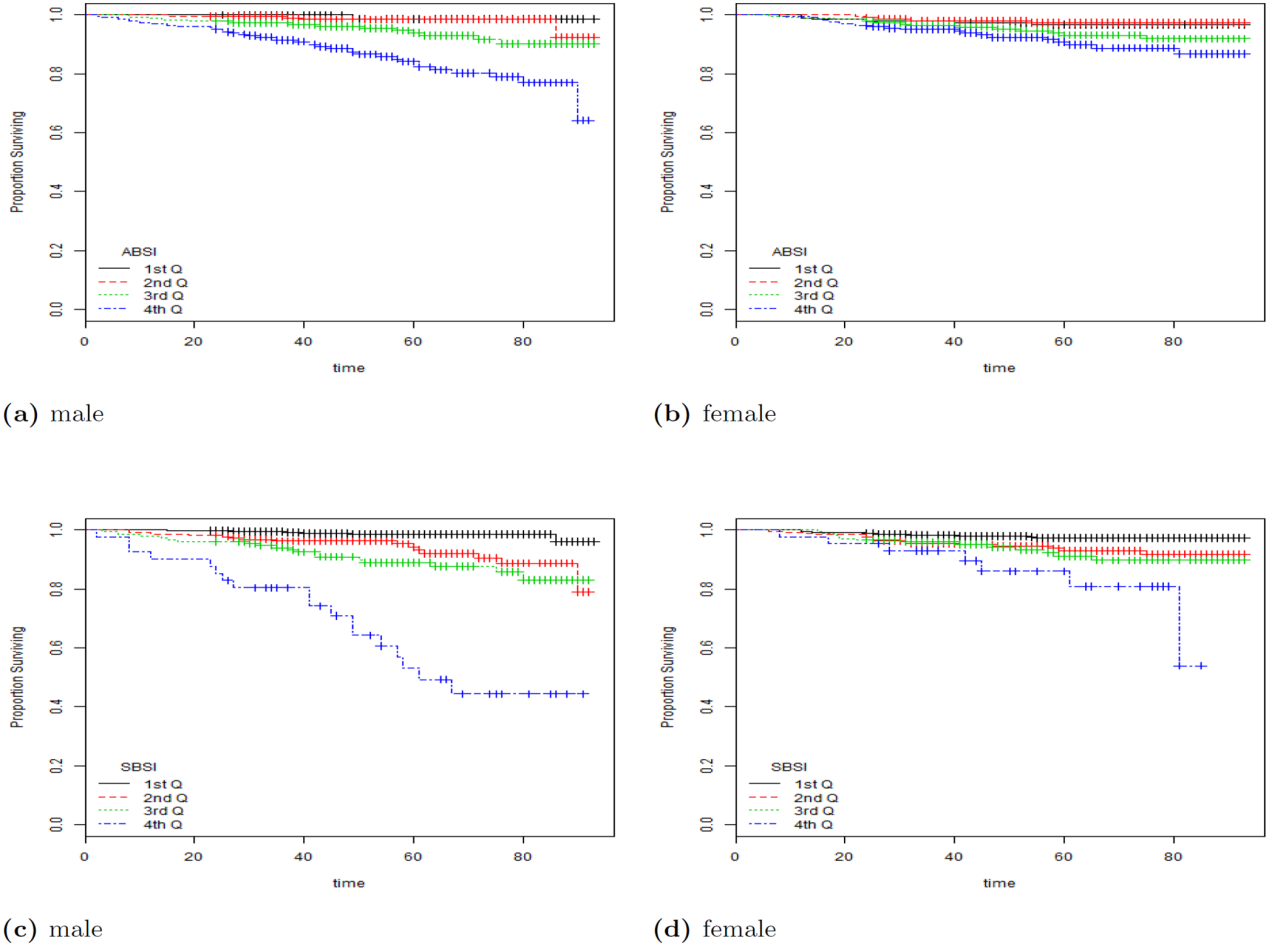
The KM curves using ABSI and SBSI on subjects in the BMI category obese I. a: ABSI (male); b: ABSI (female); c: SBSI (male); d: SBSI (female).

Fig 4 shows the detailed Kaplan-Meier curves for SBSI and three other key anthropometric body shape indices. A given variable is a good mortality predictor if the Kaplan-Meier curves are easily distinguishable (more distance between them), and the variable gives a reasonable performance from low to high levels, with less crossing between curves. SBSI performs very well in distinguishing the proportion of survivors over time (months) since examination. From the figure, it is clear that ABSI and SBSI are much better than WC and BMI in predicting survival, with the SBSI being slightly better than ABSI. The difference between ABSI and SBSI is more evident using quantitative measures, e.g., the *χ* ^2^-distance between their respective KM curves, as captured by the logrank test (Table 4). More detailed results using the hazard ratio with BMI categories are described below (see Table 5). The corresponding results for using the log-rank test to analyze the KM plots are given in Table 6, Figs 5 and 6.

**Table 5:** Results of Cox proportionality hazard modeling using different anthropometric measures.

	**All**		**Underweight**		**Normal weight**		**Overweight**		**ObeseI**		**ObeseII**		**ObeseIII**	
	11808		298		4756		4527		1715		412		100	
**All**	HR	P-val	HR	P-val	HR	P-val	HR	P-val	HR	P-val	HR	P-val	HR	P-val
*BMI*	0.916	0.023	0.115	0.002	0.792	0.138	0.691	0.075	0.813	0.542	0.189	0.065	0.819	0.809
*WC*	1.328	0	**2.91**	0.026	**3.067**	**0**	2.357	0	2.563	0	3.025	0.002	0.698	0.493
*W*	0.852	0	0.368	0.054	0.817	0.052	0.667	0	0.856	0.248	0.839	0.51	0.527	**0.25**
*BSA*	11	0	0.588	0.099	0.868	0.068	0.733	0	0.89	0.299	0.917	0.713	0.661	0.318
*VTC*	0.857	0	0.487	0.071	0.867	0.122	0.693	0	0.831	0.159	1.001	0.998	0.557	0.279
*ABSI*	**2.328**	**0**	1.905	0	2.431	0	2.264	0	2.404	0	3.555	0	0.914	0.83
*SBSI*	2.287	**0**	2.352	0	2.799	0	**2.601**	**0**	**2.952**	**0**	**3.97**	**0**	**1.004**	0.994
SBSI*	2.326	0	2.027	0	2.563	0	2.189	0	2.401	0	3.282	0	0.853	0.708
**Female**														
*BMI*	1.019	0.727	0.04	0	0.655	0.103	0.542	0.075	1.766	0.248	0.554	0.571	0.775	0.762
*WC*	1.338	0	0.513	0.493	2.45	0	1.962	0	**2.059**	0.005	**4.165**	0.001	0.912	0.869
*W*	0.828	0.009	0.031	0	0.321	0	0.326	0	0.667	0.156	0.749	0.541	0.685	0.546
*BSA*	0.752	0	0.156	0	0.467	0	0.491	0	0.714	0.082	0.86	0.648	0.874	0.753
*VTC*	0.782	0	0.056	0	0.368	0	0.389	0	0.619	0.056	0.952	0.913	0.739	0.626
*ABSI*	1.999	0	1.789	0.006	2.246	0	1.908	0	1.654	0.001	3.157	0	1.066	0.877
*SBSI*	**2.019**	**0**	**2.592**	**0.001**	**2.782**	**0**	**2.361**	**0**	2.004	**0**	4.086	**0**	**1.144**	**0.796**
SBSI*	1.969	0	1.878	0.049	2.333	0	1.892	0	1.697	0.001	3.43	0	1.041	0.925
**Male**														
*BMI*	0.823	0	0.311	0.455	0.804	0.276	0.779	0.337	0.428	0.079	0.017	0.038		
*WC*	1.254	0	**3.595**	0.016	**3.114**	**0**	2.729	0	3.581	0	3.147	0.104		
*W*	0.698	0	0.217	0.025	0.547	0	0.479	0	0.536	0.007	0.657	0.446		
*BSA*	0.68	0	0.438	0.025	0.618	0	0.554	0	0.581	0.002	0.506	0.032		
*VTC*	0.732	0	0.276	0.016	0.709	0.008	0.578	0	0.572	0.012	1.012	0.982		
*ABSI*	**2.682**	**0**	2.006	0	2.561	0	2.7	0	4.055	0	**4.9**	**0.004**		
*SBSI*	2.456	0	2.027	**0**	2.813	0	**2.781**	**0**	**4.511**	**0**	4.08	0.006		
SBSI*	2.396	0	1.732	0	2.411	0	2.318	0	3.584	0	3.69	0.005		

Results are shown for all subjects, females only, males only, and for different BMI categories. Results for the obese III category are not very reliable, as the number of data points were relatively low 0.85%, N = 100 (female 73, male 27). Notice that the P-values for this group is generally more than 0.05 for all cases. We included the results for completeness. Bold font indicate those with the best values. $SBSI^{*}≜\frac{H^{2}WC}{BSA\;VTC}$, see section on Discussion.

**Table 6:** Results using the log-rank test on the KM curves for different anthropometric measures.

	All		Underweight		NormalWeight		Overweight		ObeseI		ObeseII		ObeseIII	
	11808		298		4756		4527		1715		412		100	
**All**	*χ* ^2^-di	P-val	*χ* ^2^-di	P-val	*χ* ^2^-di	P-val	*χ* ^2^-di	P-val	*χ* ^2^-di	P-val	*χ* ^2^-di	P-val	*χ* ^2^-di	P-val
*WC*	59.465	0	0.305	0.959	153.059	0	58.597	0	11.813	0.008	1.288	0.732	0.06	0.99
*WT*	12.571	0.006	0.823	0.844	5.065	0.167	21.63	0	0.499	0.919	0.618	0.892	0.03	0.99
*BSA*	18.531	0	0.904	0.825	1.862	0.602	19.303	0	1.206	0.752	1.273	0.736	0.61	0.89
*VTC*	16.994	0.001	0.229	0.973	1.814	0.612	26.066	0	1.745	0.627	0.894	0.827	**3.78**	**0.28**
*ABSI*	551.126	0	**34.098**	**0**	**356.806**	**0**	147.489	0	53.747	0	18.375	0	0.09	0.99
*SBSI*	**570.045**	**0**	11.947	0.008	296.451	0	**202.795**	**0**	**130.434**	**0**	**43.603**	**0**	1.08	0.78
SBSI*	509.009	0	22.948	0	293.144	0	128.251	0	130.434	0	27.759	0	1.35	0.71
**Fem**														
*WC*	22.507	0	0.123	0.989	16.834	0.001	14.021	0.003	5.947	0.114	0.665	0.881	0.08	0.99
*WT*	6.926	0.074	0.123	0.989	10.973	0.012	29.139	0	0.545	0.909	0.2	0.978	0.08	0.99
*BSA*	14.943	0.002	0.42	0.936	14.599	0.002	22.985	0	2.838	0.417	0.704	0.872	0.13	0.98
*VTC*	11.18	0.011	0.036	0.998	16.017	0.001	20.318	0	8.485	0.037	0.162	0.983	0.04	0.99
*ABSI*	141.697	0	4.554	**0.208**	90.793	0	36.866	0	13.307	0.004	11.657	0.009	0.09	0.99
*SBSI*	**147.688**	**0**	4.398	0.222	**105.964**	**0**	**53.344**	**0**	**22.202**	**0**	18.08	**0**	0.36	0.94
SBSI*	135.758	0	**5.847**	0.119	81.397	0	38.768	0	14.715	0.002	**18.864**	0	**4.25**	**0.23**
**Male**														
*WC*	25.961	0	0.199	0.978	108.953	0	48.633	0	10.759	0.013	0.099	0.992		
*WT*	41.747	0	0.199	0.978	15.622	0.001	32.002	0	6.752	0.08	0.099	0.992		
*BSA*	50.449	0	0.404	0.939	19.212	0	30.106	0	8.382	0.039	0.292	0.962		
*VTC*	30.91	0	0.492	0.921	6.043	0.11	19.114	0	14.401	0.002	0.192	0.979		
*ABSI*	415.644	0	**23.068**	**0**	**251.83**	**0**	130.799	0	47.054	0	9.609	0.022		
*SBSI*	**434.372**	**0**	9.419	0.024	190.348	0	**156.804**	**0**	**124.224**	**0**	**15.347**	**0.002**		
SBSI*	420.395	0	22.986	0	210.401	0	117.32	0	67.773	0	13.571	0.004		

Results are shown for all subjects, females only, males only, and for different BMI categories. Results for the obese III category are not very reliable, as the number of data points were relatively low 0.85%, N = 100 (female 73, male 27). We can notice that the P-values for this group is generally more than 0.05 for all the cases. We included the results for completeness. Bold font indicate those with the best values. $SBSI^{*}≜\frac{H^{2}WC}{BSA\;VTC}$, see section on Discussion.

### Mortality Hazard using SBSI on BMI Categories

To further investigate the performance of SBSI in mortality modeling, we considered the mortality hazard ratio (HR) using the SBSI for each BMI category. Table 5 shows the results. In general, the hazard ratios using SBSI does not necessarily increase monotonically with increasing BMI values (BMI increases from underweight category to obese III). For instance, considering all subjects, mortality hazard ratio increased from 2.352 (*P* < 0.0001) for the underweight category, to 2.799 (*P* < 0.0001) for normal weight, and then decreased to 2.601 (*P* < 0.0001) for overweight, increasing again to 2.952 (*P* < 0.0001) for obese I. A similar trend is observed for female-only, and for male-only subjects. The table also shows the corresponding mortality hazard ratios using various other anthropometric shape indices. From the table, ABSI and the proposed SBSI tend to provide the best performance in most cases, followed by WC. Though ABSI provided the overall best result using all subjects (ABSI: HR 2.328, *P* < 0.0001; SBSI: HR 2.287, *P* < 0.0001), when split into BMI categories, SBSI provided a better performance over ABSI for all the BMI categories. SBSI was the overall best on each BMI category, except for underweight and normal weight categories (which had WC as the best performer). Using all subjects, ABSI performed better than SBSI on male subjects (ABSI: HR 2.682, *P* < 0.0001; SBSI: HR 2.456, *P* < 0.0001), while SBSI performed better on female subjects (ABSI: HR 1.999, *P* < 0.0001; SBSI: HR 2.019, *P* < 0.0001). However, again when split into BMI categories, for male subjects, ABSI outperformed SBSI only in one category–obese II (ABSI: HR 4.900, *P* = 0.004; SBSI: HR 4.080, *P* < 0.006). SBSI was the overall best in two categories, while WC was the overall best in two other categories. For female subjects, with BMI categories, SBSI provided the best results for 5 of the 7 categories, while WC reported best results for the other two categories.

To further study the performance of SBSI and other anthropometric measures using BMI categories, we analyzed the Kaplan-Meier survival curves obtained using each measure, when applied separately to subjects in each BMI category. Table 6 shows the results of this analysis. Similar to the mortality hazard, SBSI logrank result (*χ* ^2^-distance) does not increase monotonically with increasing BMI. For example, considering all subjects, the *χ* ^2^-distance increased from 11.947(P = 0.008) for the underweight category, to 296.451 (*P* < 0.0001) for normal weight and then decreased to 202.795 (*P* < 0.0001) for overweight, decreasing further to 130.434 (*P* < 0.0001) for obese I. We observe a similar trend for female-only, and for male-only subjects as well. The table also shows the results of the logrank test on other anthropometric shape indices. From the table, SBSI tends to provide the best performance in most cases followed by ABSI, WC. In general SBSI performed better than all the other anthropometric measures tested. Performing the logrank test for all subjects we get *χ* ^2^-distance 570 (*P* < 0.0001) whereas for other measures only ABSI (*χ* ^2^-distance 551 (*P* < 0.0001)) was close. For all-female (*χ* ^2^-distance 147.688, *P* < 0.0001) and all-male (*χ* ^2^-distance 434.372, *P* < 0.0001) SBSI provided overall best result. Also after splitting into BMI categories, SBSI provided the best performance as well. For all-male, all-female, and all subjects SBSI outperformed ABSI in all BMI categories except underweight (See Table 6). These results suggest that SBSI is the best anthropometric measure and distinguishes the Kaplan-Meier curves better than the other existing body measures tested.

From the results, ABSI and SBSI produced the overall best results using the KM curves on BMI categories. To further analyze the differences between ABSI and SBSI, we considered two BMI categories. Figs 5 and 6 show the KM plots for two BMI categories (overweight, and obese I), for both ABSI and SBSI. As expected, both measures indicate that female subjects generally have better survival rates when compared with male subjects. SBSI produced an overall better performance in modeling survival over time, when compared with ABSI.

## Discussion

Our proposed surface-based body shape index (SBSI) is constructed based on four key anthropometric determinants of body shape and body size: body surface area (BSA), vertical trunk circumference (VTC), height (H) and waist circumference (WC). Considered at a given height, SBSI depends on WC divided by BSA and VTC. While the BSA measures the whole body, WC and VTC measure the trunk region, with WC measured horizontally, while VTC is measured vertically. More importantly, both WC and VTC are strongly associated with abdominal fat. Previous studies [54–57] show that mortality hazard is highly related to abdominal fat. Given that SBSI has a strong association with mortality hazard, and given its definition based on WC and VTC, we suspect that SBSI will also have a significant association with abdominal fat or body volume around the waist or the trunk.

Applying SBSI initially gives reasonable performance when compared with existing body shape measures. In particular, it produced a performance that is similar to that of ABSI on most cases, and better for some BMI categories. However, given the SBSI formula, it is natural to consider some variations on the definition of SBSI. For instance, one simplification would be to remove the fractional exponents on the variables, approximating them with integral exponents. Using this, we get a simpler formula (denoted SBSI*), whose values have no units:

$$SBSI≜\frac{H^{7/4}WC^{5/6}}{BSA\;VTC}≅\frac{H^{2}WC}{BSA\;VTC}=SBSI^{*}$$ (10)

Not surprisingly, this simplified formula shows very competitive performance, producing results that are generally close to the original SBSI. See the rows denoted SBSI* in the tables (Tables 5 and 6). This competitive performance implies that SBSI* could be used in place of SBSI, depending on available computational resources, since SBSI* is just a simple unitless ratio, and easy to compute.

The measurement protocol can influence the relationship among measures for two different studies [55]. And differences between the populations involved in the study could be significant. For example, conclusions from a study based on anthropometric measurements on a Chinese population may not completely hold when applied to, say, a US population; In our work, we used two different datasets, (CAESAR and NHANES), with some differences in the way the measurements were acquired. Since we used regression parameters learned from CAESAR to apply to NHANES data, we first verified that the two data sets had similar general statistics. Participants in both studies were similar (mostly, North American, and Caucasian). The mean and standard deviation for some key attributes are as follows: height (NHANES 167.72 ± 10.1cm, CAESAR 170 ± 10.25cm), weight (NHANES 74 ± 15.8 kg, CAESAR 77 ± 19.79kg), waist circumference (NHANES 92 ± 13.23cm, CAESAR 84.77 ± 14.43cm). Thus, at least, for these key measurements, the values from the two datasets are within one standard deviation of each other.

Age is an important factor in analyzing the mortality hazard in a population. Although the SBSI generally increased with increasing age, it was still not clear exactly how age will impact the mortality hazard modeling. To further investigate this potential connection, we categorized the study population into different age ranges: < 20 (1638 people with mortality count 10), 20–35 (2937 people with mortality count 14), 36–50 (2501 people with mortality count 51), 51–70 (2678 people with mortality count 178), and > 70 (2054 people with mortality count 448). S2 and S3 Tables (Supplementary Materials) respectively, show the results for using the Cox proportionality hazard model and log-rank test on the KM curves, for mortality studies using the age categories. From the results, when using all subjects, the SBSI provided a more accurate model of the mortality hazard for people older than 35. (We ignore results for those < 20 and 20–35 group, since these groups do not contain enough mortality information). For cases of all, female-only, and male-only, SBSI performed very well on both hazard ratio and log-rank test, for the age categories 36–50, 51–70, and > 70. For the 51–70 group, for all people, the hazard ratio was 1.626. The log-rank test resulted in a *χ* ^2^-distance of 34.058 (*P* < 0.0001), almost twice as the second best ABSI (17.620, *P* < 0.0001). Thus, the KM-curves for this case is expected to be more easily distinguishable for SBSI.

In this study, we have reported results on the prediction of all-cause mortality using the proposed surface-based body shape index. We applied the index on samples from the NHANES dataset (age range 18–85), using standard BMI categories. The 11808 people in our dataset were grouped as follows: 298 underweight, 4756 normal weight, 4527 overweight, 1715 obese I, 412 obese II, 100 obese III. Our results showed that the mortality hazard as measured by SBSI (and also ABSI) does not necessarily increase monotonically with BMI. For instance, the overweight category (HR 2.264, *P* < 0.0001) showed a lower hazard ratio than the normal weight category (HR 2.799, *P* < 0.0001). Similarly, the underweight group had a lower all-cause mortality hazard (HR 2.352, *P* < 0.0001) than the normal weight group. The hazard ratio increased for obese I category (HR 2.952, *P* < 0.0001) and obese II category (HR 3.970, *P* < 0.0001). This result is consistent with previous studies on mortality hazard and BMI categories [58, 59]. We did not observe a consistent increase in mortality for increasing BMI categories. Our results using the surface-based body shape index are also consistent with previous observations of lower mortality among slightly obese and overweight groups of people [60, 61] when compared with the normal weight category. Doehner et al, [62] discussed this phenomenon, in terms of the “obesity paradox”.

### Limitations of the approach

We identify some limitations in our study. One potential problem is the lack of control for certain demographics, for instance, smoking and non-smoking status, pregnancy, socio-economic status, ancestry, etc. While these may be valid topics for future work, previous studies showed that adjusting for smoking as a variable does not significantly affect the results [63]. Similarly, pregnancy was not found to significantly impact the results.

The formulation of SBSI consists of VTC which is not available in the NHANES dataset. Thus, prediction parameters for VTC were estimated using the CAESAR dataset, and applied on subjects in the NHANES dataset. NHANES study consists of only US citizens, but CAESAR has subjects from the US, Canada, and Europe. Variability in the data collection protocols, and the general make-up of the subjects could be important sources of error. Given that both datasets are collected by trained professionals [40–43] and not self-reported, differences due to the collection protocol can be assumed to be minimal. In terms of content, both the datasets are statistically similar. For the same anthropometric attribute the average measurements from the two datasets were generally within one standard deviation of each other. See Table 1 and S1 Table (Supplementary Materials).

## Supporting Information

S1 Table Key anthropometric attributes for study participants (using the CAESAR dataset). (DOCX)

S2 Table Results for Cox Proportional Hazard applied to different age categories. (DOCX)

S3 Table Log-rank test results for different age category. (DOCX)
