# Supplementary material for: Surface-Based Body Shape Index and Its Relationship with All-Cause Mortality
Source: PLoS One. 2015 Dec 28;10(12):e0144639. doi: 10.1371/journal.pone.0144639 (PMC4692532; doi:10.1371/journal.pone.0144639)
Supplement: S1 Table — (DOCX) [file pone.0144639.s001.docx]

**Supplementary Material**

**S1 Table : Key anthropometric attributes for study participants (using the CAESAR dataset)**

|  | **All (N=11808)** | | **Female (N=5840)** | | **Male (N=5968)** | |
| --- | --- | --- | --- | --- | --- | --- |
|  | **Average** | **SD** | **Average** | **SD** | **Average** | **SD** |
| **Age (y)** | 39.621 | 12.073 | 39.821 | 12.122 | 39.396 | 12.020 |
| **Weight (W) (kg)** | 77.041 | 19.793 | 68.836 | 17.596 | 86.243 | 17.997 |
| **Height (H) (cm)** | 170.464 | 10.260 | 163.966 | 7.323 | 177.753 | 7.919 |
| **BMI (kg/m2)** | 26.351 | 5.689 | 25.582 | 6.192 | 27.214 | 4.929 |
| **Leg Length (cm)** | 53.418 | 3.989 | 50.906 | 2.828 | 56.235 | 3.127 |
| **Arm Length (cm)** | 32.591 | 2.448 | 31.381 | 2.013 | 33.949 | 2.165 |
| **Arm Circumference (cm)** | 41.723 | 5.236 | 38.782 | 4.394 | 45.021 | 3.992 |
| **Waist Circumference (cm)** | 84.801 | 14.437 | 78.896 | 13.489 | 91.419 | 12.463 |
| **Thigh Circumference (cm)** | 60.731 | 6.992 | 60.628 | 7.617 | 60.846 | 6.217 |
| **Tri-Scapular Skinfold (cm)** | 1.864 | 0.982 | 2.356 | 0.958 | 1.311 | 0.663 |
| **Subscapular skinfold (cm)** | 2.073 | 1.111 | 2.128 | 1.169 | 2.010 | 1.039 |
| **VTC (cm)** | 164.520 | 12.976 | 157.442 | 10.484 | 172.465 | 10.710 |
| **A Body Shape Index (ABSI) (m11/6kg-2/3)** | 0.074 | 0.005 | 0.071 | 0.005 | 0.076 | 0.004 |
| **Body Surface Area(BSA) (cm2)** | 18563 | 2524 | 17210 | 2025 | 20081 | 2137 |
| **WHR** | 0.497 | 0.077 | 0.482 | 0.082 | 0.514 | 0.067 |
| **Surface Based Body Shape (SBSI)** | 0.106 | 0.006 | 0.105 | 0.006 | 0.108 | 0.005 |
