# Supplementary material for: Surface-Based Body Shape Index and Its Relationship with All-Cause Mortality
Source: PLoS One. 2015 Dec 28;10(12):e0144639. doi: 10.1371/journal.pone.0144639 (PMC4692532; doi:10.1371/journal.pone.0144639)
Supplement: S2 Table — (DOCX) [file pone.0144639.s002.docx]

**Supplementary Material**

**S2 Table: Cox Proportional Hazard applied results for different age category.**

|  |  | less than20 | | 20-35 | | 36-50 | | 51-70 | | 70+ | |
| --- | --- | --- | --- | --- | --- | --- | --- | --- | --- | --- | --- |
|  |  | P-value | HR | P-value | HR | P-value | HR | P-value | HR | P-value | HR |
| ALL | BMI | 0.621 | 0.838 | 0.215 | 0.689 | 0.263 | 0.844 | 0.001 | 0.765 | 0.000 | 0.811 |
|  | WC | 0.789 | 0.906 | 0.742 | 0.911 | 0.844 | 0.971 | 0.380 | 0.931 | 0.785 | 1.014 |
|  | WT | 0.592 | **1.172** | 0.826 | 1.058 | 0.359 | 0.876 | 0.018 | 0.825 | 0.012 | 0.878 |
|  | BSA | 1.321 | 0.344 | 1.298 | 0.297 | 0.910 | 0.505 | 0.867 | 0.071 | 0.927 | 0.125 |
|  | VTC | 0.804 | 1.081 | 0.880 | 1.041 | 0.255 | 0.846 | 0.019 | 0.826 | 0.009 | 0.879 |
|  | ABSI | **0.541** | 0.784 | 0.585 | 1.167 | 0.047 | 1.406 | 0.000 | 1.544 | 0.000 | 1.504 |
|  | SBSI | 0.967 | 1.015 | **0.140** | **1.476** | **0.003** | **1.609** | **0.000** | **1.626** | **0.000** | **1.584** |
|  |  |  |  |  |  |  |  |  |  |  |  |
| Female | BMI | 0.584 | 0.608 | 0.582 | 0.714 | 0.613 | 1.111 | 0.316 | 0.891 | 0.191 | 0.908 |
|  | WC | 0.851 | 0.852 | 0.819 | 0.871 | 0.250 | 1.304 | 0.928 | 1.012 | 0.511 | 1.053 |
|  | WT | 0.571 | 0.553 | 0.721 | 0.779 | 0.761 | 1.080 | 0.293 | 0.860 | 0.058 | 0.841 |
|  | BSA | 0.653 | 0.593 | 0.881 | 0.832 | 1.042 | 0.861 | 0.867 | 0.261 | 0.845 | 0.030 |
|  | VTC | 0.599 | 0.608 | 0.581 | 0.681 | 0.697 | 1.104 | 0.140 | 0.808 | 0.018 | 0.815 |
|  | ABSI | 0.501 | 1.606 | 0.643 | 1.275 | **0.068** | **1.611** | 0.006 | 1.415 | 0.000 | 1.408 |
|  | SBSI | **0.393** | **1.884** | **0.358** | **1.630** | 0.252 | 1.355 | **0.000** | **1.558** | **0.000** | **1.480** |
|  |  |  |  |  |  |  |  |  |  |  |  |
| Male | BMI | 0.817 | 0.911 | 0.236 | 0.646 | 0.036 | 0.632 | 0.000 | 0.659 | 0.000 | 0.700 |
|  | WC | 0.751 | 0.875 | 0.566 | 0.820 | 0.069 | 0.673 | 0.027 | 0.773 | 0.014 | 0.827 |
|  | WT | 0.846 | **1.071** | 0.573 | 0.834 | 0.016 | 0.594 | 0.000 | 0.660 | 0.000 | 0.682 |
|  | BSA | 1.117 | 0.737 | 0.968 | 0.910 | 0.621 | 0.014 | 0.687 | 0.000 | 0.000 | 0.719 |
|  | VTC | 0.897 | 0.952 | 0.588 | 0.839 | **0.005** | 0.547 | 0.001 | 0.690 | 0.000 | 0.669 |
|  | ABSI | **0.427** | 0.673 | 0.503 | 1.333 | 0.540 | 1.164 | 0.000 | **1.634** | 0.000 | 1.528 |
|  | SBSI | 0.802 | 0.896 | **0.098** | **1.871** | 0.005 | **1.720** | **0.000** | 1.611 | **0.000** | **1.641** |
