# Supplementary material for: Surface-Based Body Shape Index and Its Relationship with All-Cause Mortality
Source: PLoS One. 2015 Dec 28;10(12):e0144639. doi: 10.1371/journal.pone.0144639 (PMC4692532; doi:10.1371/journal.pone.0144639)
Supplement: S3 Table — (DOCX) [file pone.0144639.s003.docx]

**Supplementary Material**

**S3 Table: Log-rank test results for different age category**

| logrank |  | **less than20** | | **20-35** | | **36-50** | | **51-70** | | **70+** | |
| --- | --- | --- | --- | --- | --- | --- | --- | --- | --- | --- | --- |
|  |  | χ^2^-dist | **p-value** | **χ^2^-dist** | **p-value** | **χ^2^-dist** | **p-value** | **χ^2^-dist** | **p-value** | **χ^2^-dist** | **p-value** |
| ALL |  |  |  |  |  |  |  |  |  |  |  |
|  | WC | **3.633** | **0.304** | 3.742 | 0.291 | 2.229 | 0.526 | 9.099 | 0.028 | 1.215 | 0.749 |
|  | WT | 0.333 | 0.954 | 6.729 | 0.081 | 1.241 | 0.743 | 10.108 | 0.018 | 5.466 | 0.141 |
|  | BSA | 1.558 | 0.669 | 4.152 | 0.246 | 1.200 | 0.753 | 4.618 | 0.202 | 1.817 | 0.611 |
|  | VTC | 0.670 | 0.880 | **7.522** | **0.057** | 1.360 | 0.715 | 11.625 | 0.009 | 8.555 | 0.036 |
|  | ABSI | 2.230 | 0.526 | 0.473 | 0.925 | **9.701** | **0.021** | 17.620 | 0.001 | 43.016 | 0.000 |
|  | SBSI | 0.419 | 0.936 | 2.371 | 0.499 | 7.957 | 0.047 | **34.058** | **0.000** | **72.415** | **0.000** |
| Female |  |  |  |  |  |  |  |  |  |  |  |
|  | WC | 2.608 | 0.456 | **3.882** | **0.275** | 2.155 | 0.541 | 4.156 | 0.245 | 2.443 | 0.486 |
|  | WT | 1.087 | 0.780 | 1.029 | 0.794 | 1.249 | 0.741 | 1.537 | 0.674 | 5.169 | 0.160 |
|  | BSA | 2.308 | 0.511 | 1.010 | 0.799 | 3.869 | 0.276 | 1.930 | 0.587 | 5.574 | 0.134 |
|  | VTC | 1.002 | 0.801 | 3.360 | 0.339 | 0.951 | 0.813 | 6.921 | 0.074 | 5.524 | 0.137 |
|  | ABSI | **5.074** | **0.166** | 0.981 | 0.806 | **4.353** | **0.226** | 7.948 | 0.047 | 15.671 | 0.001 |
|  | SBSI | 2.973 | 0.396 | 3.778 | 0.286 | 0.753 | 0.861 | **13.878** | **0.003** | **25.310** | **0.000** |
| Male |  |  |  |  |  |  |  |  |  |  |  |
|  | WC | **5.564** | **0.135** | **3.470** | **0.325** | 6.139 | 0.105 | **29.560** | **0.000** | 4.620 | 0.202 |
|  | WT | 3.019 | 0.389 | 1.690 | 0.639 | 10.413 | 0.015 | 26.433 | 0.000 | 22.360 | 0.000 |
|  | BSA | 2.672 | 0.445 | 3.002 | 0.391 | 5.759 | 0.124 | 14.895 | 0.002 | 15.869 | 0.001 |
|  | VTC | 1.952 | 0.582 | 1.831 | 0.608 | **12.047** | **0.007** | 16.346 | 0.001 | 21.869 | 0.000 |
|  | ABSI | 0.868 | 0.833 | 0.646 | 0.886 | 7.371 | 0.061 | 8.086 | 0.044 | 18.652 | 0.000 |
|  | SBSI | 2.924 | 0.404 | 3.005 | 0.391 | 9.530 | 0.023 | 18.186 | 0.000 | **34.137** | **0.000** |
